# Supplementary material for: Electronic paddle-wheels in a solid-state electrolyte
Source: Nat Commun. 2024 Jan 2;15:121. doi: 10.1038/s41467-023-44274-z (PMC10762152; doi:10.1038/s41467-023-44274-z)
Supplement: Supplementary file 1 — Supplementary Information [file 41467_2023_44274_MOESM1_ESM.pdf]

Supplementary Information
Electronic paddle-wheels in a solid-state electrolyte

Harender S. Dhattarwal,[1] Rahul Somni,[1] and Richard C. Remsing[1,*]
[1]*Department of Chemistry and Chemical Biology, Rutgers University, Piscataway, NJ 08854*

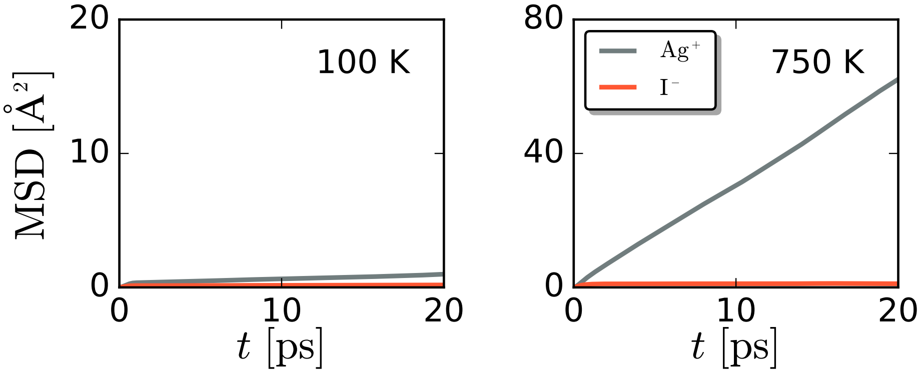

Supplementary Figure 1: Mean square displacement (MSD) of the Ag^+ and I^- ions at 100 K and 750 K.

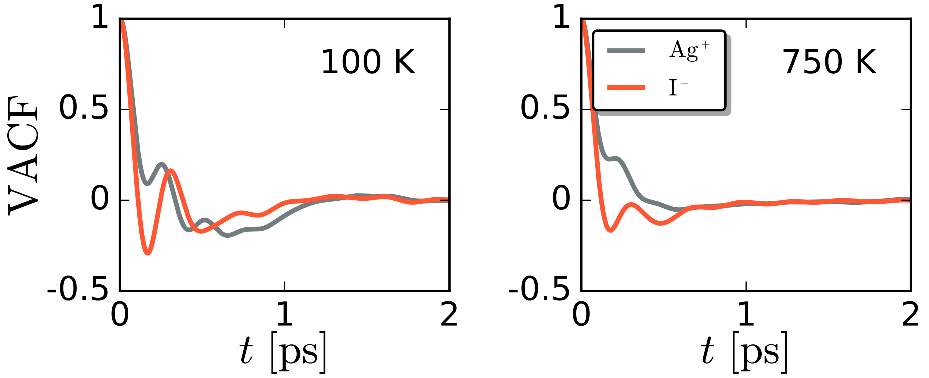

Supplementary Figure 2: Velocity autocorrelation function (VACF) of the Ag^+ and I^- ions at 100 K and 750 K.

* rick.remsing@rutgers.edu

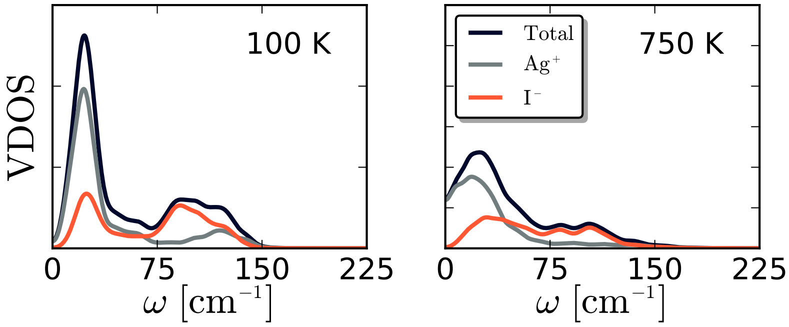

Supplementary Figure 3: The contribution of the Ag^+ and I^- ions to the Vibrational density of states (VDOS) at 100 K and 750 K.
